# Distinct Temporal Stages of Infant Brain Processing Associate With Early Versus Later Autism Diagnosis

**DOI:** 10.1111/desc.70244

**Published:** 2026-07-06

**Authors:** Tessel Bazelmans, Tony Charman, Mark H. Johnson, Emily J. H. Jones, the BASIS Team

**Affiliations:** ^1^ Department of Psychology Institute of Psychiatry, Psychology & Neuroscience King's College London London UK; ^2^ Centre for Brain and Cognitive Development Birkbeck, University of London London UK; ^3^ Department of Psychology University of Cambridge Cambridge UK; ^4^ MRC Centre for Developmental Neurobiology and Department of Child and Adolescent Psychiatry Institute of Psychiatry, Psychology & Neuroscience King's College London London UK

**Keywords:** autism, diagnosis, face processing, infant, longitudinal, mid‐childhood

## Abstract

The expression of autism traits sufficient to meet criteria for a diagnosis can occur early (by 3 years) or later (from mid‐childhood onwards). It remains unknown whether variation in age of onset is due to clinical recognition or reflects distinct biological pathways. One way of addressing this question is by investigating biological differences very early in development associated with a later age of diagnosis. We use a prospective family history design to look at event‐related potentials to faces, one of the most robust biomarkers in autism. A sample of 102 infants (aged 6–10 months, 54% female) with an older autistic sibling had an EEG recorded whilst viewing faces (faces vs. noise; gaze toward vs. away). Autism diagnostic assessments were conducted at 3 years and again in mid‐childhood (aged 6–12 years), resulting in early diagnosed (at age 3; *N* = 22), later diagnosed (at mid‐childhood; *N* = 21), and no autism in early or mid‐childhood (*N* = 59) groups. We found that while early‐stage visual processing (P100) does not associate with autism outcome, speed of structural face‐versus‐noise processing (N290) is slower in early‐onset autism only, and semantic processing (P400) is altered in both early‐ and later‐onset autism. Thus, temporal stages of face processing in infancy differentially associate with age of autism onset such that an earlier age of diagnosis is associated with earlier stage deviation within the event‐related waveform. Early and later onset autism may represent different subtypes, challenging the view of one etiological pathway and that variation in diagnostic age is solely due to clinical ascertainment.

**Summary:**

- Temporal stages of face processing in infancy differentially associate with age of autism onset.
- N290 is slower in early‐onset autism, indicating an earlier stage neural deviation.
- The later occurring P400 is altered in both early‐ and later‐onset autism.
- Early and later‐onset autism may represent distinct biological subtypes.

## 1 Introduction

Autism spectrum disorder is a neurodevelopmental condition that affects 1%–2% of children (Maenner et al. 2023; Roman‐Urrestarazu et al. 2021). There is extensive heterogeneity across the autism spectrum both between individuals with a diagnosis and within individuals across the lifespan (Lord et al. 2022). This diversity may in part reflect underlying causal genetic and/or brain differences (Jeste and Geschwind 2014; Zhang et al. 2025). Consequently, there is growing interest in stratification of autism into sub‐types that may better represent underlying biology, with resulting implications for improving early identification and support (Li et al. 2025; Lombardo et al. 2019; Loth et al. 2016; Mandelli et al. 2024; Ren et al. 2023).

One emerging potential stratification factor for autism is age of onset, which might explain some of the heterogeneity (or “chronogeneity”—heterogeneity in relation to the dimension of time) (Georgiades et al. 2017), in both core and co‐occurring trait expression (Zhang et al. 2025). Although a reliable diagnosis can be made in some children from 2 years (Ozonoff et al. 2015), the age at which autism traits manifest can vary from toddlerhood to mid‐childhood and beyond; diagnosis of autism is not made “until social demands exceed limited capacities” (DSM‐5) (American Psychiatric Association 2013). In clinical cohorts, some children are only given an autism diagnosis after 6 years despite having undergone earlier expert developmental evaluations (Davidovitch et al. 2023; Loth et al. 2016), although some received other diagnoses (e.g., language or other developmental delays, ADHD) at the earlier preschool evaluation. In population cohorts, many children do not receive a diagnosis until they reach school age (Hosozawa et al. 2020), and some may not receive a community diagnosis until adulthood (Russell et al. 2021). However, age of onset is difficult to precisely define in community samples because there is usually a delay between parents and teachers reporting difficulties and receiving a diagnosis of autism (Hosozawa et al. 2020).

Contextual factors that impact access to services, variation in local practice and under‐recognition of autism contribute to variation in age of community diagnosis (Hosozawa et al. 2020; O'Nions et al. 2023). Some variance in diagnostic timing is clearly due to societal factors or interactions with other child characteristics including gender, ethnicity, IQ, and the presence of co‐occurring neurodevelopmental conditions such as language and motor development and ADHD (“diagnostic overshadowing”; Davidovitch et al. 2023). Females tend to be diagnosed later than males (Daniels and Mandell 2014; Huang et al. 2021) and children with intellectual disability are often diagnosed earlier (Huang et al. 2021). Children from more socioeconomically deprived backgrounds have a later mean age of diagnosis (Durkin et al. 2017; Fountain et al. 2011), and there are ethnic disparities in age of diagnosis (Durkin et al. 2017; Maenner et al. 2023). Nevertheless, these factors individually account for a modest proportion of the variance in age at autism diagnosis (Zhang et al. 2025). Thus, other factors must influence the age at which autism is recognized. To identify whether the temporal manifestation of autism is truly a stratification factor requires prospective longitudinal studies in which autism traits are systematically assessed at multiple age points from infancy.

Prospective longitudinal studies of “infant sibs” (babies with a family history of autism) offer such a viewpoint by studying infants with an autism family history from early in life (Jones et al. 2014; Szatmari et al. 2016). Family history studies have shown that around 20% of infant sibs meet criteria for autism by age 3 years, consistent with the highly heritable nature of autism (Ozonoff et al. 2024). Recently, a handful of studies have followed these infants through to mid‐childhood (Bazelmans et al. 2024; Brian et al. 2016; Ozonoff et al. 2018; Shephard et al. 2017). Brian et al. (2016) and Ozonoff et al. (2018) reported that infant siblings were only given an autism diagnosis in mid‐childhood despite having undergone earlier diagnostic assessments at 3 years. In Ozonoff et al. (2018), while half had subthreshold clinical traits at the 3‐year assessment but were not given a diagnosis, half presented with no clear traits at 3 years. In a recent study, we found that twice as many children with an increased familial likelihood for autism were diagnosed with autism at mid‐childhood compared to 3 years (Bazelmans et al., 2024). Of those later diagnosed, whilst some had a mixed pattern of sub‐threshold traits on autism measures at 3 years, approximately half showed no traits and had no reported parental concerns at the earlier assessment (see Table S7 in Bazelmans et al., 2024), similar to Ozonoff et al. (2018)). Thus, there is a considerable proportion of infants with a family history of autism who, in prospective studies, do not clearly manifest autism traits until later in development.

Prospective infant family history studies offer a unique perspective through which to understand patterns of the later diagnosis of autism and whether this is “later recognition” (autism being missed earlier) or true “later emergence.” In contrast to the clinical and population studies reviewed above (Davidovitch et al. 2015, 2023; Hosozawa et al. 2020; Lord et al. 2006; Russell et al. 2021), repeated developmental and behavioral assessments—including autism diagnostic assessments using well‐established measures at the age of 3 years—are undertaken with all research participants, regardless of any expressed parental concern (or lack thereof). This circumvents the service access and eligibility issues and reduces the diagnostic overshadowing that may account for later diagnosis in clinical and population studies (Daniels and Mandell 2014; Durkin et al. 2017; Gupta et al. 2025).

Interpreting data from prospective studies also requires consideration of the degree to which family history cohorts are comparable to clinical cohorts. Although some studies have found increased parental concern regarding their younger infant child's development from 12 months of age in parents with an older child with an autism diagnosis (Ozonoff et al. 2009), others have not (Cleary et al. 2024). In our study, some parents indicated that school entry and the need to navigate complex out‐of‐home situations led to difficulties becoming more apparent (Bazelmans et al., 2024), in line with DSM‐5 (“until social demands exceed limited capacities”). In common with other prospective infant family history studies, children with autism in our cohort have higher developmental ability, the sex ratio is less skewed to males than in wider population samples, and volunteer families are biased to higher income and higher education participants (Micheletti et al. 2020; Sacrey et al. 2017). At the mid‐childhood assessment children with a later versus earlier diagnosis had lower ADI and SCQ scores (which partially capture developmental history), slightly lower total ADOS scores but comparable SRS scores (which largely capture current autistic traits), IQ and adaptive function (Bazelmans et al., 2024); this is a comparable pattern to children with earlier versus later autism profiles in a recent large‐scale study of population and diagnosis‐first cohorts (Zhang et al. 2025). Later diagnosis was more common in girls in our cohort (Bazelmans et al., 2024) as in the broader clinical literature (Dalsgaard et al. 2020). Further discussion of these clinical issues is beyond the scope of the current study but is available elsewhere (Bazelmans et al. 2024; Brian et al. 2016; Lord 2018; Ozonoff et al. 2018; Sacrey et al. 2017).

One approach to testing whether early versus later diagnosed autism within an infant sibling cohort, in part, reflects partially distinct neurobiological pathways, is to examine early brain responses. Infant sib studies have identified a range of facets of infant brain development that differ in children who later receive an autism diagnosis at 2–3 years (Dawson et al. 2023; Szatmari et al. 2016). Responses to faces have received close attention, given the relevance of early manifestations of changes in social behaviors like joint attention and gaze to eyes, for autism diagnosis. For example, electrophysiological differences in response to faces are already present in infants with a family history of autism who meet autism criteria as toddlers (Elsabbagh et al. 2012; Jones et al. 2016; Tye et al. 2022). Event‐related potentials (ERP) consist of a series of deflections representing coordinated neural activity that reliably occurs time‐locked to stimulus presentation, reflect different stages of information processing, and develop in order from lower to higher processing levels over early development (Whitehead et al. 2019). Early ERPs such as the P100 (a positive‐going deflection over the visual areas that occurs after around 100 ms) primarily reflect sensory processing. Mid‐latency components, such as the N170 in adults or N290 in infants (negative going deflections over temporal channels at the indicated latencies) reflect more structural decoding and show greater sensitivity to faces than objects or other nonsocial stimuli (de Haan et al. 2003). Longer latency components, such as the P400, reflect later stage processing (de Haan et al. 2003). The most replicated electrophysiological marker of autism is a slower N170 response to faces (Kang et al. 2018; Mason et al. 2022). In clinical samples, these atypicalities in face processing have been observed from the age of 3 (Dawson et al. 2005, 2023). Within prospective studies from infancy, infants diagnosed with autism at 3 years showed altered N290 latencies to faces versus noise relative to other infants (Tye et al. 2022), which were associated with reduced socialization skills (Tye et al. 2022) and a higher autism polygenic score (Gui et al. 2021). Further, 6‐month‐old infants with a diagnosis at 3 years showed faster P400 responses to faces compared to those without a diagnosis; shorter P400 latency was associated with more autism traits at 24 months (Jones et al. 2016). In response to dynamic gaze shifts, 8‐month‐old infants diagnosed with autism at age 3 years showed longer P100 and P400 latencies to gaze shifting towards versus away in infancy, whereas the No‐autism and typical likelihood children showed the opposite pattern (Elsabbagh et al. 2012; Tye et al. 2022). Thus, neural responses to faces in infancy are altered in children with an early diagnosis of autism.

In the current study we investigate whether infant neural responses to faces are sensitive to diagnostic timing in a prospective cohort, to test the proposal that different timing of trait manifestation represents different biological etiologies. We took the approach of focusing on a limited number of preregistered features because we were aware of the modest sample size and the likelihood for false positives if we had surveyed a broader range of EEG features. Specifically, we examine (and preregistered) infant face processing ERP responses in those children in our extended prospective family history study (Bazelmans et al., 2024) who did not receive a diagnosis until mid‐childhood (Later‐autism). We compare this Later‐autism group to children who did not receive an autism diagnosis in the timeframe of the research study (No‐autism) to determine whether there are any detectable infant neural face processing differences in children with a later diagnosis, and to those diagnosed at age 3 years (Early‐autism) to assess whether there are differences in infant neural face processing related to diagnostic timing. Of note, we have already examined the relation of infant ERP responses to 3‐year diagnosis in this cohort (Gui et al., 2021; Tye et al., 2022). Specifically, based on previous work, we preregistered hypotheses regarding the modulation of the latency of the N290 to faces versus noise (Gui et al., 2021; Tye et al., 2022) and modulation of the latency of the P100 and P400 to gaze shifting toward versus away from the infant (Gui et. al., 2021). If early‐ versus later‐diagnosed cases represent distinct subgroups, they should significantly differ from each other and from children without a later diagnosis on these components. Last, we hypothesize that any infant ERP features that associate with later diagnosis (e.g., shorter N290 to face vs. noise and longer P100 and P400 to gaze toward vs. away) should be associated with higher social communication and restricted and repetitive autistic traits in mid‐childhood given evidence of shared etiology between dimensional traits and categorical diagnosis (Robinson et al. 2016; Ruzzo et al. 2019) (for additional details on our hypotheses, also see pre‐registration (link below) and Supporting Information, available online).

## 2 Methods and Materials

The hypotheses and analysis plan for this manuscript are pre‐registered on Open Science Framework (OSF). We have noted in the text where we have deviated from the pre‐registered analysis plan. Results from the pre‐registered alpha‐connectivity data are mentioned briefly in Section 3. Details of methods, analyses and findings can be found in Supporting Information 2. We do not center these findings, given that associations with categorical diagnosis of autism were not clear in the original papers on this data (Haartsen et al., 2019), but have included these results for completeness.

### 2.1 Participants

**FIGURE 1:**
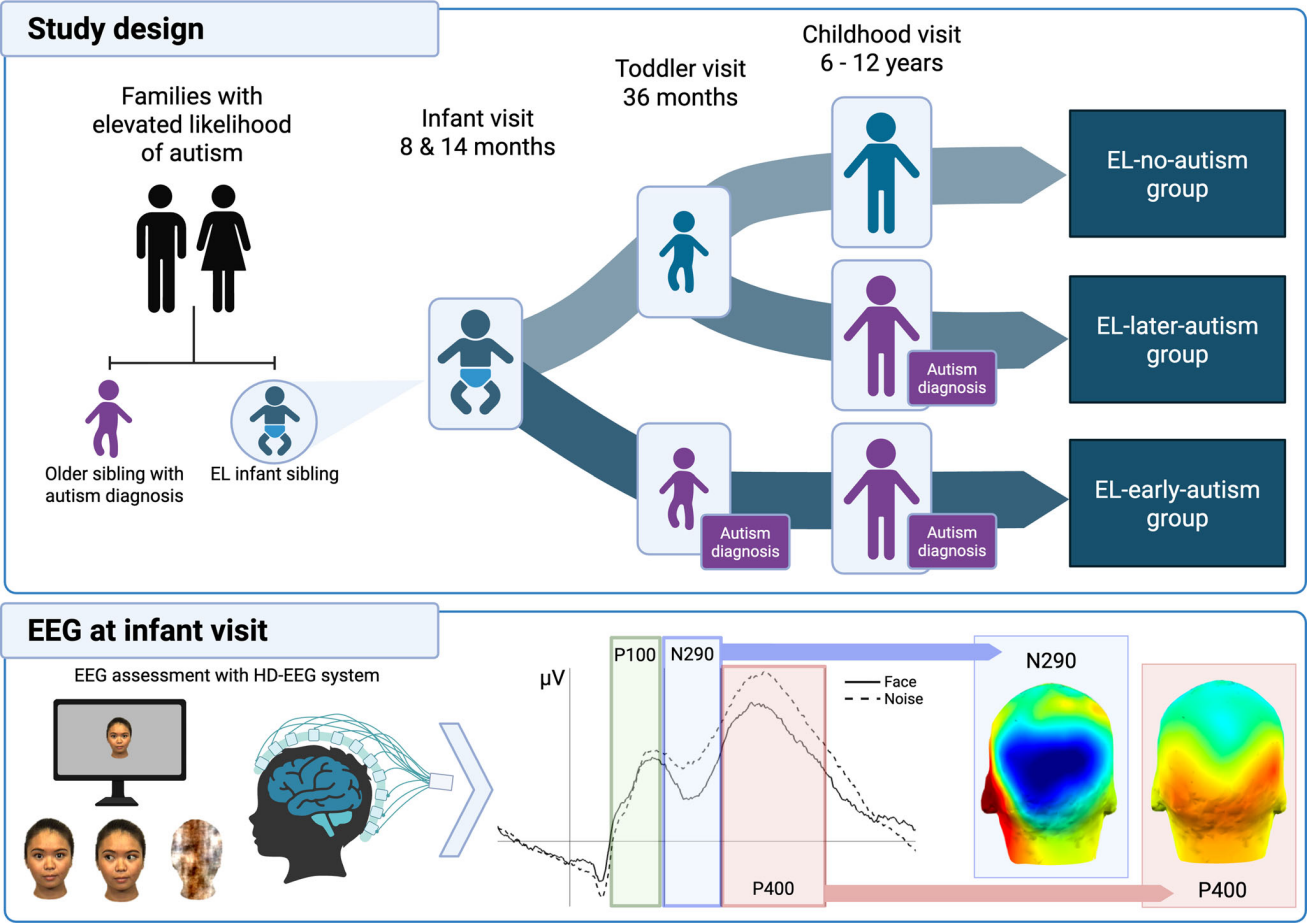
Study design and stimuli.

The data are from Phase 1 and 2 of the longitudinal British Autism Study of Infant Siblings (BASIS; www.basisnetwork.org) (see Figure 1 for study design), which follows infants with an elevated likelihood of autism (because of an older sibling with a diagnosis of autism) and those with an older sibling but without a first‐degree family member with autism (typical likelihood) through multiple timepoints in early development (8‐, 14‐, 24‐, and 36‐months). Families were recruited from urban and rural areas across the United Kingdom and visits took place in London. We followed 159 elevated likelihood infants up in mid‐childhood (6 years 9 months–12 years 8 months; Mean: 8 years 11 months) (Bazelmans et al., 2024). We excluded three infants because they received a research diagnosis at 3 years, but not at mid‐childhood (Shephard et al., 2017) of the remaining children, 102 had EEG data at infancy and a diagnostic outcome at mid‐childhood available: 59 (22 male) No‐autism, 22 (15 male) Early‐autism, and 21 (10 male) Later‐autism (Gender: *χ* ^2^ (2) = 6.18, p = 0.045). To note, we repeated all analyses, excluding two children with only a half‐sibling with a diagnosis, to align with the pre‐registration, but results remained the same.

#### 2.1.1 Electrophysiological Measures (8 Months)

The task was previously reported (Elsabbagh et al., 2012; Tye et al., 2022) (also see Supporting Information 3) and was designed to assess electrophysiological responses to: (1) faces (static and irrespective of gaze direction) versus visual noise (N290) and (2) dynamic gaze shifts (gaze toward vs. away from the infant; P100 & P400; see Figure 1 for stimuli). EEG was recorded from a 128 channel Hydrocel Sensor Net. All preprocessing and ERP computations were done offline in EGI Net Station version 5.2.0.2. Data was filtered from 0.1 to 100 Hz and epoched from –200 to 800 ms. Trials were retained only when the infant was fixating on the center of the screen at stimulus onset, with no gaze shifts, blinks or head movements during the remainder of the epoch. After baseline correction (–200 to 0), we used automatic artifact detection followed by manual channel and epoch rejection through visual inspection. Up to 12 bad channels were interpolated and otherwise the trial was rejected. Data was average referenced and averaged across epochs with a minimum of 10 valid trials per infant per condition. The mean number of trails available in each condition ranged from 21 to 61. There were no significant differences between groups on the number of total and valid trials on any of the conditions (Supporting Information 3, Table S2). Peak amplitude and latency of the averaged P100, N290, and P400 across occipitotemporal channels for each stimulus/contrast were used for subsequent analyses (Elsabbagh et al., 2012; Tye et al., 2022).

#### 2.1.2 Clinical Assessments (Age 3 Years and Mid‐Childhood)

Experienced researchers and clinicians (including TB, TC) determined the best estimate clinical outcome at both 3 years and mid‐childhood by reviewing all available information from visits using ICD‐10 (Phase 1: 3 years) or DSM–5 (Phase 1: mid‐childhood and Phase 2) criteria.^1^ This information included (but was not limited to) the Autism Diagnostic Interview—Revised (ADI‐R) (Lord et al. 1994), Autism Diagnostic Observation Schedule (ADOS) (Gotham et al. 2009), Vineland Adaptive Behavior Scale 2nd (VABS‐II) (Sparrow et al. 2005) or 3rd edition (VABS‐3) (Sparrow et al. 2016), Mullen Scales of Early Learning (Mullen 1995) (24 and 36 months), and Wechsler Abbreviated Scale of Intelligence (WASI) (Wechsler 2011) (mid‐childhood). Researchers were not blind to either likelihood group or to 36 months best estimate clinical outcome (see Bazelmans et al., 2024 for details).

#### 2.1.3 Social Communication and Restricted Interest and Repetitive Behaviors (Mid‐Childhood)

Parents reported on their child's autism traits by completing the Social Responsiveness Scale (SRS‐2) (Constantino and Gruber 2012). This 65‐item questionnaire was rated on a 4‐point Likert scale, ranging from not true to almost always true. The two main subscales were calculated: the Social Communication and Interaction (SCI; 53 items) and the Restricted Interests and Repetitive Behavior (RRB; 12 items) subscales.

### 2.2 Statistical Analysis

All analysis were performed in Stata 18 (StataCorp 2023). Mixed models were run (*mixed* command, followed by *contrast* command), including the random intercept for participant ID, using restricted maximum likelihood (REML). Based on previous findings, our hypotheses and analyses are focused on the latency models; amplitude models can be found in Supporting Information 4 and showed no significant group or interaction effect. In the first model, diagnostic outcome (No‐autism, Early‐autism, Later‐autism), condition (face vs. noise/toward vs. away) and their interaction effect were included (example code: *mixed p100_latency i.Group##i.Cond || id*:). Taking account of our two‐Phase cohort design and some of the factors that community studies have shown can relate to age of diagnosis, the models were repeated, including Phase, gender, age, and non‐verbal developmental score (NVT) at the 8‐month visit as covariates. We also repeated the analyses, including mid‐childhood WASI scores (not pre‐registered; see Section 3 and Supplement 5). Model fit of the mixed models was checked by inspecting the normality of standardized residuals, and models were repeated excluding any standardized residuals >|3|. Any significant interaction was further explored by comparing groups using the *margins Outcome, dydx(condition) pwcompare* command. Our hypotheses rest on the contrasts between the Later‐autism and Early‐autism/No‐autism groups and are False Discovery Rate (FDR) corrected for these two comparisons (as per pre‐registration). An approximate effect size was calculated by estimating the additional explained variance of significant predictors using the r2_mlm command (Rights and Sterba 2019). This calculates the change in total variance explained by fixed effects (marginal R^2^) in models including and excluding the significant variable. We then converted this into Cohen's *f* (Selya et al. 2012). The contrast Early‐autism versus No‐autism is reported for completeness and because in previous reports the No‐Autism group included some children who in the current study were given a later mid‐childhood autism diagnosis, but not at the 3‐year assessment (Tye et al., 2022), but was not corrected for, considering this was not a contrast of interest. For completeness, we show the EEG data for the control typical likelihood (TL) group in Supporting Information 6.

To examine dimensional bivariate associations between infant ERP (P100, P400, and N290 difference scores; calculated as, for example, latency for face toward—face away condition)) and mid‐childhood autism traits (SRS‐SCI and SRS‐RRB), we ran separate Kendall Tau_b_ correlations for the sample as a whole, followed by separate correlations for the No‐autism group and the combined Later and Early‐autism groups for any significant associations. We planned to conduct multivariate regression, but due to the skewness of the SRS data, these models did not lead to a good fit, even after transformation, and so we used separate non‐parametric tests instead and corrected for multiple comparisons using FDR (*n *= 6 for latency difference scores). Significant correlations were repeated as ordinal regressions with SRS scores as outcome variable, including also Phase, gender, and age and NVT at the infant visit. Dimensional associations with the ADI‐R and ADOS‐2 scores are reported in Supporting Information 7.

## 3 Results

**FIGURE 2:**
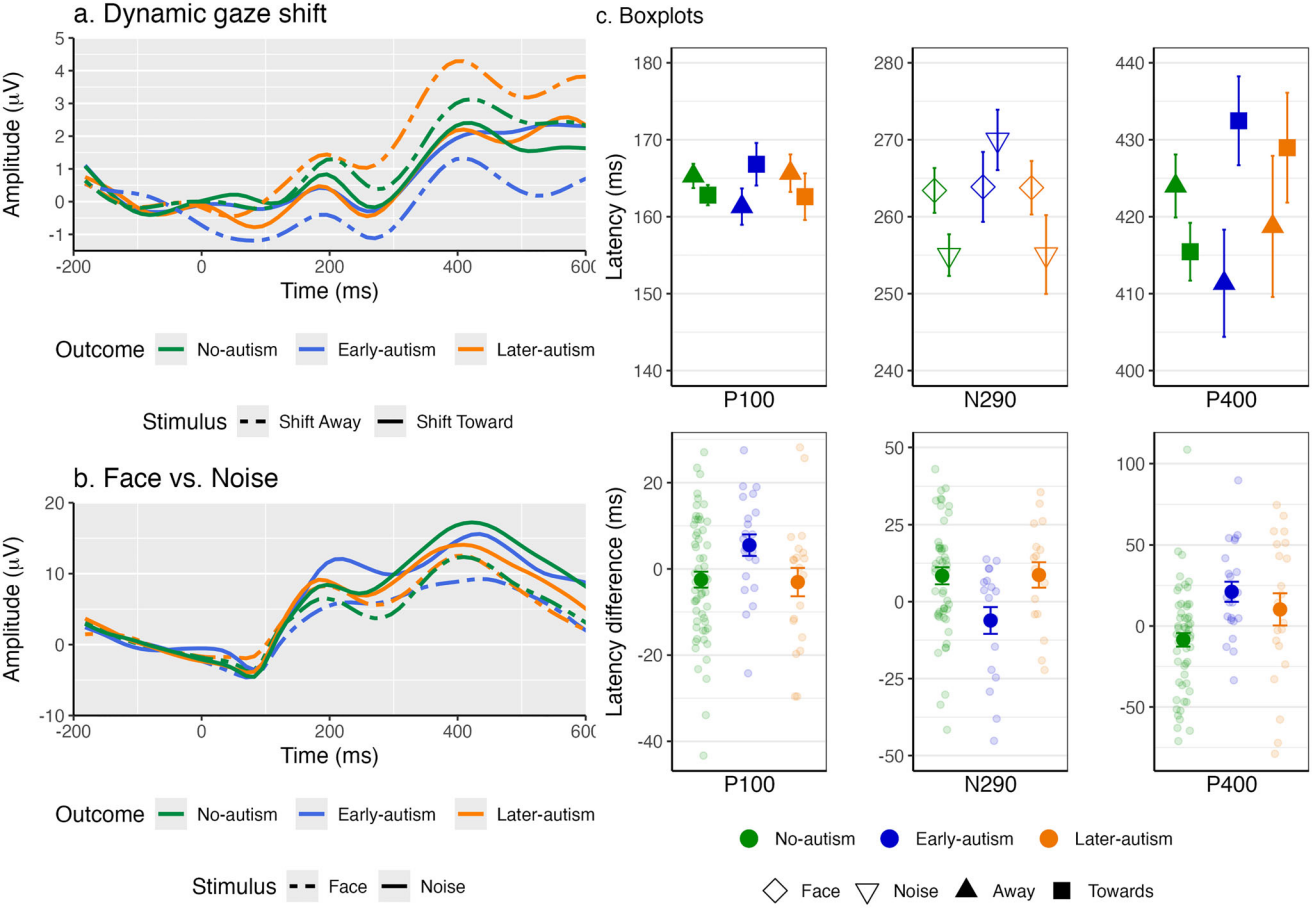
Grand average ERP waveforms for (a) dynamic gaze shifts and (b) face versus noise. (c) Boxplots including Mean and SE of latency responses by ERP and mid‐childhood outcome group.

Descriptive statistics can be found in Table 1. As previously reported (Bazelmans et al., 2024), the Later‐autism group showed lower Vineland and higher SRS scores at mid‐childhood compared to the No‐autism group, but similar to the Early‐autism group (indicating no clear differences in “severity”/autistic trait level based on early vs. later diagnosis). ERP descriptive can be found in Table 2 and is visualised in Figure 2.

**TABLE 1:** Descriptive statistics by outcome group.

	Elevated likelihood autism outcome									
	No‐autism (*N* = 59)			Early‐autism (*N* = 22)			Later‐autism (*N* = 21)			
	*N*	%		*N*	%		*N*	%		*X* ^2^ (*p*)
Phase										3.69 (0.158)
1	23	39%		10	45%		4	19%		
2	36	61%		12	55%		17	81%		
Sex										6.18 (0.045)
Male	22	37%		15	68%		10	48%		
Female	37	63%		7	32%		11	52%		
Ethnicity										1.06 (0.589)
										
										
Asian/Black African/ Black Caribbean/Mixed, etc.	9	15%		5	23%		5	24%		
										
White/European/Irish etc.	50	85%		17	77%		16	76%		
Annual household income										1.46 (0.481)
Up to £40,000	22	38%		8	38%		5	24%		
Over £40,000	36	62%		13	62%		16	76%		
8 Months	M	(SD)	*n*	M	(SD)	*n*	M	(SD)	*n*	*F* (*p*)
Age (months)	8.27	(1.20)	59	8.18	(1.10)	22	8.24	(1.18)	21	0.05 (0.954)
Non‐verbal *t*‐score	52.92	(9.57)	59	50.50	(11.00)	22	56.76	(11.38)	21	2.04 (0.135)
Mullen ELC	100.98	(13.39)	59	97.95	(18.92)	22	107.10	(16.74)	21	2.00 (0.141)
Vineland ABC	91.61	(14.57)	57	90.18^a^	(15.81)	22	100.14^b^	(10.97)	21	3.36 (0.039)
Mid‐childhood										
Age (months)	106.80	(17.28)	59	105.36	(16.89)	22	113.86	(16.94)	21	1.63 (0.201)
WASI FSIQ	108.61	(13.01)	59	107.76	(18.34)	21	107.76	(18.77)	21	0.04 (0.964)
Vineland ABC	101.77^a^	(10.96)	57	87.50^b^	(15.34)	20	89.70^b^	(13.58)	20	13.19 (<0.001)
SRS SCI total	26.89^a^	(19.47)	54	68.44^b^	(33.22)	18	60.06^b^	(31.97)	17	23.72 (<0.001)
SRS RRB total	3.65^a^	(4.81)	54	15.17^b^	(11.39)	18	11.41^b^	(7.12)	17	21.50 (<0.001)

*Note*: Different superscript (a, b) differs significantly after Tukey HSD pairwise comparison.

**TABLE 2:** 8‐Month‐old infant ERP latencies by mid‐childhood outcome group.

	No‐autism (*N* = 59)			Early‐autism (*N* = 22)			Later‐autism (*N* = 21)		
	M	(SD)	*n*	M	(SD)	*n*	M	(SD)	*n*
P100 latency									
Shift towards	162.81	(10.11)	59	166.82	(12.97)	22	162.60	(13.89)	21
Shift away	165.30	(12.12)	59	161.31	(11.08)	22	165.66	(11.16)	21
Towards‐away	−2.49	(14.17)	59	5.51	(11.71)	22	−3.06	(15.07)	21
N290 latency									
To faces	263.41	(20.81)	51	263.87	(19.28)	18	263.77	(14.33)	17
To noise	255.01	(19.33)	51	269.98	(16.64)	18	255.09	(21.12)	17
Faces‐noise	8.40	(19.81)	51	−6.11	(18.43)	18	8.68	(17.03)	17
P400 latency									
Shift towards	415.45	(28.57)	58	432.46	(27.08)	22	428.97	(32.69)	21
Shift away	423.99	(31.18)	58	411.35	(32.67)	22	418.74	(42.03)	21
Towards‐away	−8.54	(31.80)	58	21.11	(29.10)	22	10.23	(45.52)	21

*P100 Latency*: The overall model was not significant, indicating that group and condition did not explain significant variance (Wald *χ* ^2^ (5) = 6.38, *p* = 0.271). The model became marginally significant after adding mid‐childhood WASI scores (*p* = 0.056) and the WASI score itself was significant in the model (*B* = 0.14, SE = 0.06, *p* = 0.017; also see Supporting Information 5).

*N290 Latency*: The overall model was significant (Wald *χ* ^2^ (5) = 18.31, *p* = 0.003). There was no main effect of group (*χ* ^2^ (2) = 2.97, *p* = 0.227) or condition (*χ* ^2^ (1) = 2.48 *p* = 0.115) but there was a significant group by condition interaction effect (*χ* ^2^ (2) = 8.36, *p* = 0.015). Of the 6.53% of variance explained by all fixed effects, 2.20% was attributable to the interaction effect (Cohen's *f* = 0.024, small effect). To investigate the interaction effect, we compared groups on the face versus noise contrast. This showed a significant difference between the Later‐autism and Early‐autism groups (contrast = –14.79, SE = 6.43, *p* = 0.022, 95% CI [–27.40, –2.18], after FDR correction: *p* = 0.044): the Later‐autism group had longer latencies to face versus noise compared to the Early‐autism group who showed longer latencies to noise versus face. The Later‐autism and No‐autism groups did not differ (contrast = –0.28, SE = 5.33, *p* = 0.958, 95% CI [–10.72, 10.16]). As previously reported (Tye et al., 2022)[ , the Early‐autism and No‐autism groups significantly differed from each other (contrast = 14.51, SE = 5.22, *p* = 0.005, 95% CI [4.29, 24.73]). None of the control variables were significant predictors (all *p* > 0.094).

*P400 Latency*: The overall model was significant (Wald *χ* ^2^ (5) = 14.02, *p* = 0.016). There was no main effect of group (*χ* ^2^ (2) = 0.40, *p* = 0.818) but there was a significant effect of condition (*χ* ^2^ (1) = 3.95, *p* = 0.047) and a group by condition interaction (*χ* ^2^ (2) = 13.34, *p* = 0.001). Of the 4.1% of variance explained by all fixed effects, 3.8% was attributable to the interaction effect (Cohen's *f* = 0.040, small effect). Pairwise comparison of groups on the toward versus away contrast showed a significant difference between the Later‐autism and No‐autism group (contrast = –18.77, SE = 8.79, *p* = 0.033, 95% CI [–36.01, –1.54], after FDR correction: *p* = 0.066). The Later‐autism group had longer P400 latencies to faces shifting toward versus away, compared to the No‐autism group, who showed the opposite pattern. The Later‐autism group did not differ from the Early‐autism group (contrast = 10.88, SE = 10.53, *p* = 0.302, 95% CI [–9.77, 31.53]). As previously reported (Tye et al., 2022), the Early‐autism group also showed longer P400 latencies to faces toward versus away compared to the No‐autism group (contrast = –29.66, SE = 8.65, *p* = 0.001, 95% CI [–46.60, –12.71]). Results became more pronounced in the later versus no‐autism groups after removing one outlier that that was observed after plotting the contrast score (>3SD above their group mean; see also Supporting Information 3, Figure S2): Later‐autism versus No‐autism: contrast: –20.83, SE = 8.31, *p* = 0.012, after FDR correction: *p* = 0.024, 95% CI [–37.12, –4.54). Mid‐childhood WASI score was significantly associated with P400 latency when added to the model (*B* = 0.34, SE = 0.17, *p* = 0.042); none of the other control variables were significant.

*Associations with autism traits*: There was a significant association between P400 latency difference score in infancy and both social‐communication impairment (Ktau_b _= 0.193, *p = *0.008, after FDR correction: *p *= 0.024; No‐autism: Ktau_b _= –0.087, *p *= 0.369, Early+Later: Ktau_b _= 0.221, *p = *0.050) and restricted and repetitive behavior (Ktau_b _= 0.214, *p *= 0.004; corrected: *p *= 0.024; No‐autism: Ktau_b _= –0.046, *p *= 0.650, Early+Later: Ktau_b _= 0.219, *p *= 0.055) SRS subscales in mid‐childhood. Specifically, slower responses to gaze turning toward versus away were associated with higher autistic traits in mid‐childhood. These associations with the P400 latency remained significant (SCI: *B* = 0.014, SE = 0.005, *p* = 0.007; RRB: *B* = 0.015, SE = 0.005, *p* = 0.007) after including the control variables (all *p* > 0.243). Removing the same outlier as in the P400 latency group comparison models did not change the results. The associations between P400 and ADOS and ADI scores were also significant (*p* ≤ 0.050, Supporting Information 7). There was a significant association between N290 latency and ADI Social (Ktau_b _= –0.187, *p *= 0.015) and RRB scores (Ktau_b_ = –0.176, *p* = 0.031). No other associations were found between the mid‐childhood SRS, ADOS, or ADI scores and either the P100 or N290 latency difference scores in infancy.

*Alpha‐Connectivity*: There was no effect of group (*p *= 0.236), there was an association with mid‐childhood RRB traits as reported on the ADI (Ktau_b_ = 0.190, *p *= 0.032; No‐autism: *N* = 42, Ktau_b_ = 0.082, *p *= 0.51, Early+Later: *N* = 29, Ktau_b _= 0.312, *p *= 0.024) (Supporting Information 2).

## 4 Discussion

This is the first study to identify differential infant brain processing markers for children who received an early versus later diagnosis of autism, and the first to show that there are differences in the infant brain in children who are diagnosed with autism after toddlerhood. We compared ERP responses to faces of 8‐month‐old infant sibs with an autism diagnosis in toddlerhood versus in mid‐childhood, and infants without autism at mid‐childhood. We found that different stages of brain processing of faces in infants differentially associate with the age of trait manifestation. Specifically, while a short latency component (P100) did not differentiate between elevated likelihood groups, mid‐latency face processing (N290) was different only in children with early diagnosis, and a longer latency face processing component (P400) was different in children with both early and later diagnosis, relative to infants without an autism diagnosis. The pattern of findings did not change when cohort (Phase), gender, age, and non‐verbal developmental ability at the 8‐month assessment were accounted for.

### 4.1 Later‐Autism Similar to Early‐Autism Group at Later Stage Processing

We tested whether the Later‐autism group differed on ERP latencies from the No‐autism group, hypothesizing that underlying brain differences would already be evident in infancy, even if phenotypic expressions did not (yet) meet diagnostic criteria by the age of 3 years. The Later‐autism group responded similarly to the Early‐autism group but differently from the No‐autism group for P400 latency, showing that the Early‐ and Later‐autism groups have similar neurodevelopmental differences in later‐stage face processing. We also found dimensional associations between the latency of the infant P400 response and mid‐childhood autism trait severity both for social communication difficulties and for restricted, repetitive and rigid behaviors, suggesting that later, mid‐childhood trait measures of autism severity, as well as categorical autism diagnosis, are associated with differences in later stage electrophysiological components. However, the Later‐autism group were significantly different from the Early‐autism group on the latency of the earlier N290 component, instead resembling children who did not meet criteria for autism at either timepoint (No‐autism). Thus, differences in early‐stage face processing are specific to autism that manifests earlier in development.

### 4.2 Parallel Between Brain Processing and Developmental Manifestation

The findings imply a parallel between the stage of real‐time brain processing of faces and the timing of the developmental manifestation of autism traits. The earlier behavioral traits become evident, the earlier in the ERP waveform atypicalities in information processing occur, suggesting disruptions in relatively early‐stage information processing. Differences in those with and without an autism diagnosis at age 3 years became evident from early stages of face‐specific structural/configural processing onwards. The N290, mostly recorded by posterior electrodes over the temporal lobe area like the adult N170, is thought to reflect the encoding of structural information in faces (de Haan et al. 2003; Halit et al. 2003). We have previously shown that infants with Early‐autism show faster N290 responses to face versus noise at 8 months (Gui et al., 2021; Jones et al., 2016; Tye et al., 2022). The N290 is considered a precursor of the adult N170 response, which has repeatedly been found to be delayed in autism (Kang et al. 2018). Notably, while both infants and children with autism show alterations in the N290/N170, the direction reverses from *faster* N290 responses to faces versus noise in infancy to *slower* N170 responses in children and adults; the reason for this transition is not known but other social attention phenotypes show similar developmental reversals (e.g., more looking at eyes at 2 months vs. less looking at eyes at 2 years; Jones and Klin 2013). Given that we show that the infant N290 only varies in children with an early diagnosis, and as the N170 has been considered as a biomarker for autism (Mason et al. 2022; McPartland et al. 2020), it will be important to evaluate whether considering chronogeneity in the form of age of manifestation would improve the use of the N170 as a stratification biomarker in older cohorts.

### 4.3 Later‐Autism Differences Associated With Semantic Processing of Faces

Children who received a diagnosis of autism at mid‐childhood, but not at age 3 years, showed disruptions that only emerged at the later P400 component. The P400 occurs around 400 ms after stimulus onset and reflects mid to higher‐level, semantic information processing, and is mostly observed in posterior and lateral regions (de Haan et al. 2003). Like the Early‐autism group, the Later‐autism group showed longer P400 latencies to faces shifting gaze toward versus away, but this was opposite from the No‐autism group (a finding similar to our exploratory work (Bedford et al., 2017). Thus, these effects may suggest differences in more complex aspects of social perception in children with both early and later autism diagnosis. Notably, the observed effects were not downstream consequences of altered very early sensory perception. Specifically, we did not find any changes in P100 latency in either early or later diagnosed groups in line with other reports in infants (McCleery et al. 2009) and adults (Mason et al. 2022). The P100 is thought to reflect early sensory processing, peaking around the occipital area, and responses are usually not specific to faces in infants. Thus, whereas basic sensory processing seems relatively typical in infants with both Early and Later‐autism, issues with structural/configural face processing (N290) are present in those with early autism, and children with a later manifestation of autism traits might have particular issues with integrating the perceptual input with semantic meaning reflected by the P400 component (Li et al. 2024). Additionally, no group effects or associations with later traits on ERP amplitude were found (see Supporting Information 4 and 7). Although various studies have suggested differences in amplitude in infants later diagnosed with autism (Elsabbagh et al. 2012), these effects have not consistently been reported to be specific to later diagnosis and instead are found to relate to autism family history status (Tye et al. 2022).

### 4.4 Severity or Chronogeneity Models May Explain Heterogeneity

Together our evidence supports an intriguing association between diagnostic timing and real‐time neural processing. Our findings potentially relate to two different models underlying heterogeneity in autism: (1) a model in which older age diagnosis is due to a less penetrant (“less severe”) version of the same underlying neurodevelopmental processes as early‐onset autism, resulting in a less prominent manifestation of behavioral traits, only challenged by the greater social environment complexity encountered in mid‐childhood and possibly detected later by tools that may lack sensitivity where traits are “less severe” or in, for example, girls, sometimes described as the Unitary model (Zhang et al. 2025) and (2) a chronogeneity model in which, over and above the other factors that can affect age of diagnosis, later (vs. earlier) manifesting cases represent a distinct biological pathway with different genetic, brain, and cognitive etiologies (Kendler et al. 2024; Zhang et al. 2025). There is a range of additional emerging genetic evidence consistent with chronogeneity. Zhang et al. (2025) show that in both population and clinical samples, the age of diagnosis is itself a heritable trait, and two partially overlapping polygenic scores are associated with earlier versus later diagnoses. Convergingly, Pourcain et al. (2018) identified two polygenic traits that impact developmental variation in social and communication difficulties in the ALSPAC cohort, with one accounting for variation in these scores only in adolescence. Further, Martini et al. (2024) showed in a twin sample that only around a third of variation in autistic traits due to genetics was shared across development. Others have used data‐driven approaches to identify phenotypic clusters with different developmental onset profiles that in turn vary on genetic or neuroimaging parameters (Litman et al. 2025; Mandelli et al. 2024), or shown connectivity differences in fMRI data associated with age of diagnosis (Li et al. 2025). While our finding of different infant ERP profiles associating with different ages of manifestation is also broadly consistent with chronogeneity, we acknowledge that two aspects of our data could also be taken to support the former one. First, subtle differences in excitation/inhibition balance may lead to signal‐to‐noise changes whose effects become increasingly compounded as a sequence of neural processing proceeds (Faisal et al. 2008; Hancock et al. 2017). Smaller deviations from neurotypical brain processing may result in ERP differences that appear later in the waveform, and thus also correspond to a milder atypicality and later onset. Second, slower P400 responses (but not N290 responses) were associated with higher autistic traits in mid‐childhood in both the cohort as a whole and within children with an autism outcome only, consistent with reduced atypicality in infant late‐stage ERP responses associated with later trait severity.

## 5 Limitations

One limitation is that our data did not allow us to look more closely at the specific age traits manifested, but rather those meeting criteria at 3 years versus 6‐to‐12 years, the two timepoints at which we conducted autism diagnostic assessments. This precluded us from examining the dimensional association between the age of trait manifestation and differences in the EEG waveform timing. We also note that some children we have currently characterized as “No‐Autism” on the basis that they did not meet diagnostic criteria at either the 3‐year or the mid‐childhood diagnostic assessment might be given an autism diagnosis at a later age in adolescence or even adulthood, were such a continued follow‐up assessment to be conducted in the future. If these children shared a neurodevelopmental EEG profile with the Later‐autism group, this may mean our current P400 comparisons are an underestimate of the true effect size; given the similarity in means for the N290 contrast (8.40 vs. 8.68), and this is unlikely to become significant. Thus, although our conclusions are likely robust to the possibility of children in the No‐autism group being later diagnosed, this consideration is a reminder that both the study of autism itself and the infant family history design are fundamentally developmental studies where characterization is not static. Researchers were not blind to the child's developmental history, including whether they were assigned a research diagnosis at 3; this was because evaluation of autism requires a full developmental history, but might make it less likely that children “lost” their diagnosis within this cohort (Bazelmans et al., 2024). We also acknowledge that findings from family history “infant sibling” cohorts might not generalize to the wider population of autistic children, including individuals with non‐familial autism (Szatmari et al. 2016). In infant family history studies, children who go on to have autism are less likely to be significantly developmentally delayed, more likely to be female, and less likely to have de novo and inherited high impact genetic variants than in clinical cohorts (Litman et al. 2025; Zhang et al. 2025). While it would be of value to examine the same neural processing signatures in individuals from clinical cohorts who have an earlier or later reported age of diagnosis, in such cohorts (who have not undergone repeated prospective diagnostic evaluations) certainty around true later emergence will be less secure. Notwithstanding these challenges, examining the role of age of diagnosis in existing neuroimaging autism cohorts would be valuable and should be done.

### 5.1 Preregistration and Need for Converging Evidence

One specific choice with both advantages and limitations is our focus on one specific preregistered face ERP response. We pre‐registered our hypotheses for these specific measures to reduce the likelihood of false positives from a data‐driven approach, given the modest sample; we selected face responses as they have been replicated in the autism literature (Kang et al. 2018; Mason et al. 2022) and have shown associations with polygenic scores, an area of the literature in which most progress has been made on the chronogeneity hypothesis (Gui et al. 2021; Zhang et al. 2025). One associated limitation is that by focusing on a feature that has been previously demonstrated to be different in children with an autism diagnosis at 3, we were less likely to detect other features that may be solely associated with later diagnosis; replication in independent samples is, of course, also necessary. We did initially preregister analysis of a second EEG feature (infant EEG alpha connectivity), because of its replicable associations to dimensional phenotypes (RRBs) at age 3 within the group of infants with an autism diagnosis. As previously reported, differences in alpha‐connectivity between the no and early autism groups found in our Phase 1 cohort (Orekhova et al., 2014) were not replicated when we added our Phase 2 cohort (Haartsen et al., 2019, and re‐analysis did not identify any additional differences between the Later‐autism and Early/No‐autism groups. However, we found that our initial association between increased alpha‐connectivity within a fronto‐central mask and increased RRB traits as measured with the ADI at age 3 years was also present when RRB traits were measured in mid‐childhood. This was present in the EL group as a whole; within the children with an autism outcome and present at trend level within the later diagnosed cohort, but not the Early or No‐autism groups when analyzed separately; at 3 years, the association with connectivity was also specific to the subgroup of children who met diagnostic criteria and not present in other groups. Although preliminary, given the sample size, this suggests that differential connectivity profiles in infancy are most closely related to RRB traits within autistic children at the time of diagnosis. Finding the strongest association with 14‐month data in the age group most recently diagnosed (early or later) illustrates the importance of taking a developmental approach to such data. It remains unknown how or whether earlier versus later diagnoses will differ across other tasks; establishing chronogeneity will ultimately require converging evidence from multiple sources, including genetics (Zhang et al. 2025).

### 5.2 Specificity of Results

Equally, we do not currently know the specificity of this finding to autism relative to other neurodevelopmental conditions. Previous studies have reported on different face‐processing ERP response in children with autism, ADHD or both (Tye et al. 2013). Considering our sample consists of children with a familial likelihood for autism (and not ADHD), in addition to the elevated levels of ADHD traits within our early and later autism groups (Bazelmans et al. 2024, the current dataset is not well positioned to address the specificity of this response.

We also found that the latency of infant responses was associated with mid‐childhood WASI score. The results suggest that a longer response latency in infancy, which may reflect deeper processing, is associated with higher cognitive scores. This association was, however, not pre‐registered and further research is needed to understand the association between ERP latencies and later cognition, but it does highlight the importance of considering the cognitive abilities of the sample.

Nevertheless, our prospective study provides a unique design where we can combine infant EEG with longitudinally reported traits in a cohort in which diagnosis is not influenced by demographic or societal factors that affect the age at which community diagnoses are received from clinical services (Hosozawa et al. 2020; Russell et al. 2021).

## 6 Conclusion

To conclude, our findings on the association between stage of real‐time brain processing and age of trait onset, together with recent evidence that age of diagnosis is associated with partially distinct genetic and clinical profiles (Zhang et al. 2025), support the view that the autism spectrum could be stratified by “chronogeneity”—informative variance about the developmental time course encompassing individual trajectories of emergence (Georgiades et al. 2017). Understanding mechanistic and biological differences related to the age of onset could bring new insights and provide better targeted developmentally sensitive intervention and support for autistic individuals.

## Author Contributions

**Tessel Bazelmans**: conceptualization, investigation, data Curation, formal Analysis, writing Original Draft, writing Review Editing. **Tony Charman**: conceptualization, funding Acquisition, writing Review Editing. **Mark H. Johnson**: conceptualization, funding Acquisition, writing Review Editing. **Emily J. H. Jones**: conceptualization, funding Acquisition, writing Review Editing.

## Funding

This research was supported by awards from the Medical Research Council (MR/R011427/1, G0701484, MR/K021389/1, MR/T003057/1), BASIS funding consortium led by Autistica, Autism Speaks. The results leading to this publication have received funding from the Innovative Medicines Initiative 2 Joint Undertaking under grant agreement No 777394 for the project AIMS‐2‐TRIALS. This Joint Undertaking receives support from the European Union's Horizon 2020 research and innovation programme and EFPIA and AUTISM SPEAKS, Autistica, SFARI. Any views expressed are those of the authors and not necessarily those of the funders (IHI‐JU2). European Union Horizon Europe grant no. 101057385 (R2D2‐MH) and UK Research and Innovation (UKRI) under the UK government's Horizon Europe funding guarantee [grant no.10039383] and by the Swiss State Secretariat for Education, Research and Innovation (SERI) under contract number 22.00277. Tessel Bazelmans is supported by a grant from the UK Medical Research Council (MR/X010716/1). Views and opinions expressed are however those of the authors only and do not necessarily reflect those of the European Union. Neither the European Union nor the granting authority can be held responsible for them. Capital equipment funding from the Maudsley Charity (980) and Guy's and St Thomas’ Charity (STR130505). The funders had no role in the design of the study; in the collection, analyses, or interpretation of data; in the writing of the manuscript; or in the decision to publish the results.

## Ethics Statement

Ethical approval was obtained from the NHS National Research Ethics Service (NHS RES London REC 06/MRE02/73, 08/H0718/76 and 14/LO/0170) and PNM Research Ethics Subcommittee, King's College London (RESCM‐18/19‐10556).

## Consent

Parents gave informed consent to participate in the study.

## Conflicts of Interest

Tony Charman has served as a paid consultant to F. Hoffmann‐La Roche Ltd. and receives royalties from Sage Publications and Guilford Publications. Mark H. Johnson receives royalties from Wiley‐Blackwell, OUP, and MIT Press. The remaining authors have declared that they have no competing or potential conflicts of interest.

## Supporting information



Supporting Information: desc70244‐supp‐0001‐SuppMat.docx

## Data Availability

Data available following a review of requests as indicated here: https://www.basisnetwork.org/collaboration‐and‐project‐affiliation/index.html

## Endnotes

1 In the Phase 1 cohort 3‐year diagnostic assessments were completed using ICD‐10 but on subsequent review all cases who met criteria for a ICD‐10 pervasive developmental disorder were also considered to meet DSM‐5 criteria for autism spectrum disorder.
