# Supplementary material for: Distinct Temporal Stages of Infant Brain Processing Associate With Early Versus Later Autism Diagnosis
Source: Dev Sci. 2026 Jul 6;29(5):e70244. doi: 10.1111/desc.70244 (PMC13338452; doi:10.1111/desc.70244)
Supplement: Supplementary file 1 — Supporting Information: desc70244‐supp‐0001‐SuppMat.docx [file DESC-29-e70244-s001.docx]

**Stages of brain processing in infants associate with early versus later autism diagnosis – Supplementary Materials**

Table of Contents

[Supplement 1: Pre-registered hypotheses 2](#_Toc224049821)

[Supplement 2: Alpha-connectivity 4](#_Toc224049822)

[Supplement 3: Additional EEG methods 7](#_Toc224049823)

[Supplement 4: EEG Amplitude 11](#_Toc224049824)

[Supplement 5: Mid-Childhood WASI Scores 12](#_Toc224049825)

[Supplement 6: Typical Likelihood Group 13](#_Toc224049826)

[Supplement 7: Associations with Autism traits 15](#_Toc224049827)

[References 18](#_Toc224049828)

# Supplement 1: Pre-registered hypotheses

Our detailed hypotheses, including hypotheses related to visual disengagement and alpha-connectivity (not reported in this manuscript) were pre-registered on Open Science Framework (OSF; <https://doi.org/10.17605/OSF.IO/XSYU7>). Here we report the hypotheses related to the EEG/ERP data:

We hypothesise that the late diagnosis group has a similar early-emerging neurodevelopmental difference that underlies their eventual diagnosis of autism by mid-childhood as the early diagnosis group but that this might differ by degree or severity such that for the late diagnosis group the emerging behavioural atypicalities are insufficient for them to meet diagnostic criteria at the age of 3 years (e.g., subthreshold). In line with our hypothesis that the late diagnosis group has underlying (but less severe) early-emerging neurodevelopmental differences, we expect the EL-late-autism group to be significantly different from the EL-no-autism group (H1). Further, we test two possible predictions:

• the EL-late-autism group does not differ from the EL-early-autism group on neural/cognitive measures in infancy. That is, the early and late groups are similar at a neural/cognitive level, despite the EL-late-autism group not meeting diagnostic criteria on a behavioural level at 3 years and only doing so in mid-childhood (H2.1); or

• the EL-early-autism group has a more severe manifestation of autism terms of neural/cognitive differences compared to the EL-late-autism group. This underlies their higher behavioural atypicality that reaches the threshold for diagnosis at 3 years and their higher severity of autism symptoms in mid-childhood.

Based on this theory, we expect a graduation of difference in infancy such that the EL-late autism group will significantly differ from the EL-early-autism group. The direction would be such that the EL-late-autism group has measures closer to those without autism and the EL-early-autism group being most ‘atypical’ (H2.2).

For the ERPs specifically, we hypothesize that:

- N290 latency: (i) the EL-late-autism group will show shorter latencies to face versus noise compared to the EL-no-autism group (H1) (ii) and the EL-late-autism group will have latencies similar to the EL-early-autism group, i.e. no differentiation between noise vs face on N290 latency (H2.1) or will show longer latencies to face versus noise compared to the EL-early-autism group (H2.2)(but not as long as the EL-no-autism group).
- P100 + P400 latency: (i) the EL-late-autism group will show P100/P400 latencies that are significantly different from the EL-no-autism group, who will show longer latencies to gaze shift away versus towards (H1). (ii) and the P100/P400 latencies of the EL-late-autism group will be either similar to the EL-early-autism group, i.e. latencies are longer to gaze shift towards versus away (H2.1) or the contrast between towards versus away will be smaller compared to the EL-early-autism group (H2.2)(but still different from the EL-no-autism group).
- Hypothesis 3: Based on the observed differences in infant measure in association with outcome groups (see Hypothesis 1 & 2) and previous findings of associations with later traits, we expect the infant measures show an association with autism traits at mid-childhood. Specifically, we expect: ERP measures to be associate with traits in the social communication domain, especially faster N290 response to noise versus face and faster P100 response to shift away versus towards associated with better social skills (Tye et al., 2022) and larger N290 response to face versus noise to be associated with higher social communication problems (Shephard et al., 2020).

# Supplement 2: Alpha-connectivity

*Background:*

We also pre-registered a comparison between early and later autism diagnosis related to alpha-connectivity during social videos. The full pre-registration can be found here: <https://doi.org/10.17605/OSF.IO/XSYU7> and below we have included the relevant sections from the pre-registration:

Our previous results showed that:

“(i) based on three-year outcomes, the EL-autism group showed higher alpha-connectivity compared to the TL and EL-no-autism groups (Orekhova et al., 2014)(cohort 1). This group difference was not replicated in cohort 2 (Haartsen et al., 2019). However, hyper-connectivity associated with more RRB at 3 years in the EL-autism group (Orekhova et al., 2014), and this finding was replicated in cohort 2 (Haartsen et al., 2019).”

With regards to group differences, we hypothesized that:

“(i) the EL-late-autism group shows higher levels of alpha connectivity compared to the EL-no-autism group (H1). (ii) and the EL-late-autism group shows similar levels of alpha connectivity as the EL-early-autism group (H2.1) or significantly lower levels of alpha-connectivity compared to the EL-early autism group (H2.2)(but not as low as the EL-no-autism group).”

With regards to associations between alpha-connectivity and traits, we hypothesized that:

“Based on the observed differences in infant measure in association with outcome groups (see Hypothesis 1 & 2) and previous findings of associations with later traits (Haartsen et al., 2019; Orekhova et al., 2014) we expect the infant measures show an association with autism traits at mid-childhood.” “Specifically, we expect […] increased alpha-connectivity to associate with the RRB domain, specifically in the EL-early-autism and EL-late-autism groups (see (Haartsen et al., 2019; Orekhova et al., 2014)).”

*Methods* (also see (Haartsen et al., 2019; Orekhova et al., 2014))

14-month-old infants were presented with 3 different dynamic videos, presented two times (thus 9 videos in total). These videos were played in random order and depict spinning toys (duration: 44 s), a hand spinning the toys around (duration: 41 s), and women singing nursery rhymes (duration: 32 s). EEG was recorded whilst the infants attended the videos sittings on their parent’s lap in an electrically shielded room. The infants’ behaviour, including looking and movement, during this EEG session was recorded with a video camera and these periods were excluded from analysis. EEG was recorded using a 128-electrode HydroCel Geodesic Sensor Net (EGI, Eugene, OR)

EEG segments were excluded from further analyses when the infant was not paying attention, interference occurred, or when the segment contained artefacts. After manual and automatic artefact rejection, the remaining data segments were cut into 1-s epochs with 50% overlap. Infants with more than 120 epochs across all three conditions were included in further analyses. Fast Fourier Transform (FFT) was performed after a Hanning window was applied to each of the clean epochs. The Fourier transformed data were used for functional connectivity analyses and spectral power analyses.

Functional connectivity was measured with the debiased weighted phase lag index (dbWPLI) that was calculated for each possible pair of connections from the FFT values. Values close to 0 reflect low connectivity whereas values closer to 1 reflect high connectivity. The functional connectivity matrices were averaged across frequencies for 7–8 Hz. Global dbWPLI values were calculated by averaging dbWPLI values for all possible pairs of connections. Functional connectivity was also calculated in the selected connections by averaging dbWPLI values for those connections that separated HR-ASD infants from the control comparison groups in the first publication (Orekhova et al., 2014).

*Analysis plan:*

As pre-registered, we ran a Kruskal-Wallis with as outcome measure alpha-connectivity over fronto-central regions (as specified in Haartsen et al. 2019) at 14 months and the predictor was outcome group: No-autism, Early-autism, Later-autism). If significant, we planned to run the model as a general linear model including: gender, age at visit, cohort, NVT at 14m and spectral power in alpha-band (frontal regions). To look at the association between alpha-connectivity and traits, we again ran Kendall Tau_b_ due to distributions on the variables.

*Sample:*

In total, 42 No-autism, 14 Early-autism and 15Later-autism had valid EEG data available at 14 months. Of these, 65 also contributed to the previous ERP analysis and 6 were additionally added. Overall, the description of the alpha-connectivity sample did not differ from the main paper: no group differences were observed between phases, gender, ethnicity or income. There were also no group differences at 14 months in age and non-verbal t-score,

There was a difference in 14-month Mullen Early Learning Composite (F = 3.15, *p* = .047) and Vineland Adaptive Behavior Composite (F = 3.39, *p* = .037). The early-autism group had significantly lower scores compared to the no-autism group for the Mullen (-8.72, *p* = .041), but group comparisons were not significant after multiple-correction for the Vineland.

At mid-childhood, age and WASI scores did not differ, but the early and later autism groups had higher SRS scores on both the Social scale (F = 29.99, *p* <.001, early vs no: 42.59, *p* <.001, later vs no: 40.05, *p* <.001) and the Repetitive Behavior Scale (F = 25.19, *p* <.001, early vs no: 11.58, *p* <.001, later vs no: 9.36, *p* <.001). Vineland Adaptive Behavior Composite scores were also significant lower in both autism groups (F = 17.15, *p* <.001, early vs no: -14.45, *p* <.001, later vs no: -14.03, *p* <.001).

*Results:*

We did not find a significant effect of group (χ2 = 2.89, *p* =. 236). We did find a significant association between alpha-connectivity and ADI-RRB scale (Kendall Tau_b_ = 0.19, *p* = .032) which is in line with our previous findings. This shows that alpha-connectivity at 14 months is related to parent reported Restricted and Repetitive Behaviors in mid-childhood. Looking at associations in groups separately, the association were Kendall Tau_b_ = 0.08, *p* = .513, Kendall Tau_b_ = 0.18, *p* = .408, Kendall Tau_b_ = 0.37, *p* = .076 for no, early and later-autism, respectively, but note the low sample size in individual groups. In line with our pre-registered hypothesis, the association was also significant in the combined Early+Later groups: Kendall Tau_b_ = 0.31, *p* = .024. We did not find any other associations of alpha-connectivity with SRS and ADI subscales (see Table S1). As discussed in the main text, finding the strongest association with 14-month data in the age group most recently diagnosed (early or late) illustrates the importance of taking a developmental approach to such data.

| Table S1: *Associations between Alpha-Connectivity scores and autism traits (Kendall Tau_b_ (p))* | | | | | | | | | |
| --- | --- | --- | --- | --- | --- | --- | --- | --- | --- |
|  | SRS | |  | ADOS | |  | ADI | | |
|  | SCI | RRB |  | Social Affect | RRB |  | Social | Comm. | RRB |
| Alpha-Connectivity | .077 (.368) | .020 (.829) |  | .057 (.497) | .072 (.433) |  | .101 (.229) | .123 (.148) | **.190 (.032)** |
| Note. N=65 for SRS, 71 for ADOS/ADI. SRS = Social Responsiveness Scale; ADOS = Autism Diagnostic Observation Schedule; ADI = Autism Diagnostic Interview; SCI = Social Communication and Interaction; RRB = Restricted Interests and Repetitive Behaviour; Comm = Communication. Associations using Kendall Tau_b._ | | | | | | | | | |

# Supplement 3: Additional EEG methods

Methods are as previously reported (Elsabbagh et al., 2012; Tye et al., 2022). Infants sat on their parents’ laps at a 60 cm distance from a 40 x 29 cm computer screen. Gaze during stimulus presentation was recorded by video camera. Each trial block began with a static colorful fixation stimulus followed by a color image of one of four female faces, with gaze directed either toward or away from the infant. In subsequent trials of the same block, the face remained on the screen but displayed three to six gaze shifts, alternating from directed toward to away from the infant. Faces were aligned with the center of the screen with the eyes appearing at the same location as the fixation stimuli, to ensure that infants were fixating the eye region. The faces subtended 21 x 14 degrees of visual angle. In addition to face trial blocks, during approximately one third of all blocks, infants were presented with ‘‘visual noise’’ stimuli. The latter were constructed from the same faces presented within the task, by randomizing the phase spectra while keeping the amplitude and color spectra constant. Fixation stimuli, preceding the onset of the face and noise stimuli, subtended approximately 1.6 3 1.6 degrees and were presented for a variable duration of 800 to 1,200 ms. Each trial lasted for 1,000 ms. A 128 channel Hydrocel Sensor Net was mounted on each infant’s head, while they were seated on the parent’s lap in front of the stimulus screen. When the infant was attending toward the screen, trials were presented continuously for as long as the infant remained attentive, with brain electrical activity measured simultaneously using the vertex as a reference (Cz in the conventional 10/20 system). EGI NetAmps 200 was used (gain = 1,000). Data were digitized with a sampling rate of 500 Hz and band-pass filtered between 0.1–100 Hz.

Data were stored and analysed offline in EGI Netstation version 5.2.0.2 (using the same protocol as (Elsabbagh et al., 2012). Trials were retained only when infants were fixating on the centre of the screen at stimulus onset, without any gaze shifts, blinking or head movements during the 800ms segment following stimulus onset. Data were then corrected to the -200ms baseline. Following automated artifact detection, an experienced EEG researcher (CT). conducted detailed manual artifact rejection through visual inspection of individual trials. Data from any sensor were excluded if they contained artifacts. Missing data from 12 or fewer channels were interpolated, otherwise the entire trial was rejected. Data were then rereferenced to the average.

Stimulus-locked epochs (-200 to 800ms peristimulus window) were averaged for the following trial contrasts: 1. faces (valid static (irrespective of gaze direction) vs. visual noise stimuli presented at the beginning of each block); and 2. dynamic gaze shifts (gaze toward vs. away from the infant, after appearance of the initial face within each block). Averages were computed for each participant in each condition on a minimum of 10 trials. Due to variable rates of presentation of each stimulus type, a different number of trials were included for each contrast, which did not differ by outcome group. The occipito-temporal montages from (Elsabbagh et al., 2012) were used (Figure S1) and corroborated with visual inspection of the grand average for each condition across the three contrasts. Peak amplitude and latency of the average P100, N290 and P400 responses were included in subsequent analyses because consistently modulated in face processing tasks in the first year of life.

| 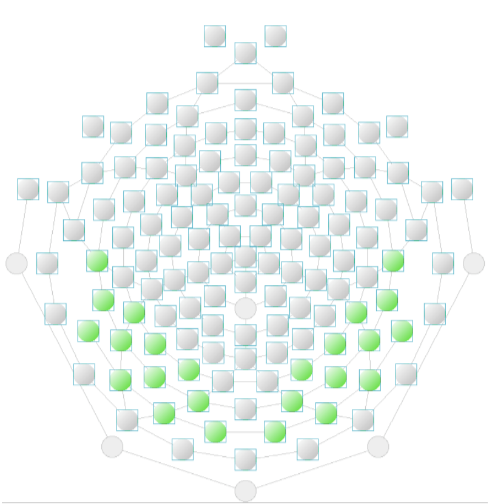 | 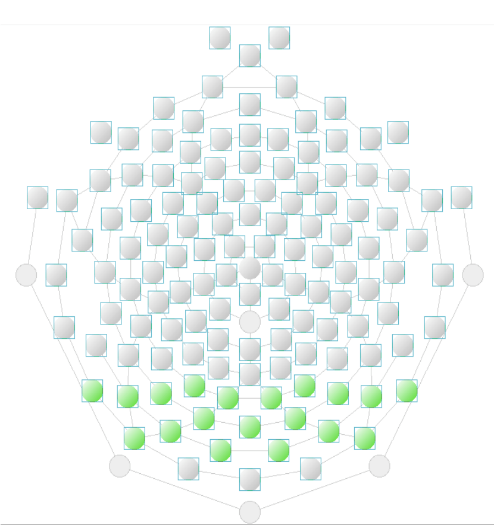 |
| --- | --- |
| 1. Dynamic gaze contrast | 1. Face/noise contrast |
| *Figure S1.* Selected channel montages based on (Elsabbagh et al., 2012) and (Tye et al., 2022) corroborated with visual inspection of grand averages | |

| Table S2: *Valid trial numbers for ERP analysis by outcome group and by phase* | | | | | | | | | |
| --- | --- | --- | --- | --- | --- | --- | --- | --- | --- |
|  |  | | **TL** | **EL** | | | | **ANOVA F (p)** | |
| **Phase** |  |  |  | **All** | **No** | **Early** | **Later** | **EL only** | **All** |
|  | *Total N* |  | 43 | 99 | 59 | 21 | 20 |  |  |
| **1^a^** | *Gaze shift N:* | | *31* | *34* | *22* | *9* | *3* |  |  |
|  | Toward | Trials | 124.77 | 125.65 | 135.95 | 105.67 | 110 | 0.60 (.090) | 1.89 (.141) |
|  |  | Valid | 55.74 | 61.65 | 68.73 | 42.33 | 67.67 | 2.42 (.105) | 1.82 (.152) |
|  | Away | Trials | 124.87 | 124.00 | 134.23 | 104.11 | 108.67 | 2.53 (.096) | 1.81 (.154) |
|  |  | Valid | 55.97 | 61.95 | 69.64 | 40.56 | 69.67 | 3.06 (.061) | 2.26 (.090) |
|  | *Face/noise N:* | | *23* | *29* | *19* | *7* | *3* |  |  |
|  | Face | Trials | 67.22 | 65.79 | 69.74 | 60 | 54.33 | 2.67 (.085) | 1.92 (.136) |
|  |  | Valid | 37.91 | 37.10 | 42 | 25 | 34.33 | 3.45 (.045) | 2.34 (.081) |
|  | Noise | Trials | 47.83 | 44.45 | 46 | 42 | 40.33 | 0.79 (.463) | 1.02 (.389) |
|  |  | Valid | 23 | 29.00 | 19 | 7 | 3 | 1.73 (.194) | 1.18 (.323) |
| **2** | *Gaze shift N:* | | *12* | *65* | *36* | *12* | *17* |  |  |
|  | Toward | Trials | 104.08 | 105.72 | 105.75 | 105.08 | 106.12 | 0.01 (.993) | 0.02 (.996) |
|  |  | Valid | 39.17 | 51.77 | 50.53 | 60.75 | 48.06 | 1.26 (.291) | 2.05 (.115) |
|  | Away | Trials | 105.5 | 105.72 | 106.22 | 106.17 | 104.35 | 0.04 (.961) | 0.03 (.994) |
|  |  | Valid | 38 | 51.20 | 49.83 | 59.75 | 48.06 | 1.15 (.322) | 2.18 (.097) |
|  | *Face/noise N:* | | *10* | *55* | *31* | *10* | *14* |  |  |
|  | Face | Trials | 54.9 | 56.09 | 55.81 | 56.8 | 56.21 | 0.04 (.964) | 0.04 (.989) |
|  |  | Valid | 21.7 | 30.15 | 27.97 | 36.5 | 30.43 | 1.35 (.267) | 2.46 (.069) |
|  | Noise | Trials | 40.1 | 41.56 | 41.42 | 41.8 | 41.71 | 0.08 (.928) | 0.06 (.982) |
|  |  | Valid | 18.4 | 21.87 | 20.68 | 25.5 | 21.93 | 0.75 (.475) | 1.01 (.393) |
| *^a^ Number of Total and Valid trials missing for 1 infant in each EL group in Phase 1* | | | | | | | | | |


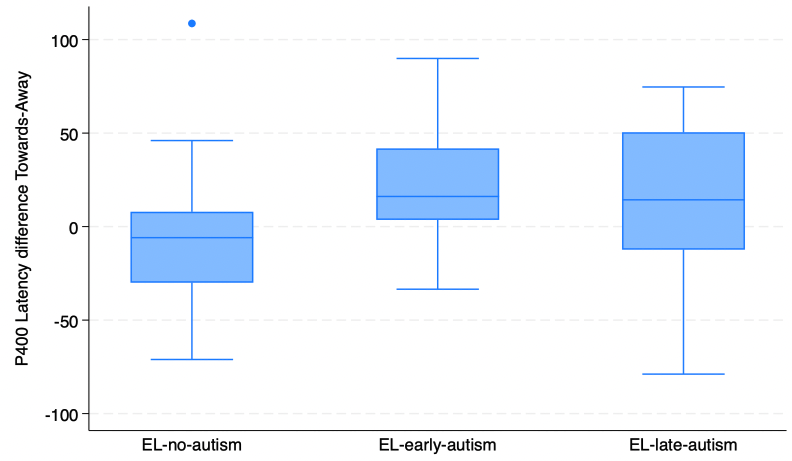


*Figure S2.* Boxplot of P400 Latency contrast, showing outlier (>3SD) in No-autism group.

# Supplement 4: EEG Amplitude

| Table S3: *8-month-old infant ERP amplitude by mid-childhood outcome group* | | | | | | | | | |
| --- | --- | --- | --- | --- | --- | --- | --- | --- | --- |
|  | No-autism (N=59) | | | Early-autism (N=22) | | | Later-autism (N=21) | | |
|  | M | (SD) | n | M | (SD) | n | M | (SD) | n |
| P100 Amplitude Shift Towards | 0.61 | (2.43) | 59 | 0.41 | (2.81) | 22 | 0.31 | (2.51) | 21 |
| P100 Amplitude Shift Away | 0.97 | (2.44) | 59 | 0.14 | (3.30) | 22 | 1.07 | (1.91) | 21 |
| P100 Amplitude Towards-Away | -0.36 | (2.76) | 59 | 0.27 | (4.24) | 22 | -0.76 | (2.87) | 21 |
| N290 Amplitude to Faces | 4.40 | (6.86) | 51 | 5.69 | (6.41) | 18 | 5.83 | (6.34) | 17 |
| N290 Amplitude to Noise | 8.93 | (6.99) | 51 | 10.83 | (6.78) | 18 | 7.62 | (4.52) | 17 |
| N290 Amplitude Faces-Noise | -4.54 | (7.42) | 51 | -5.14 | (7.80) | 18 | -1.79 | (7.04) | 17 |
| P400 Amplitude Shift Towards | 1.86 | (3.75) | 59 | 2.02 | (3.25) | 22 | 1.89 | (3.84) | 21 |
| P400 Amplitude Shift Away | 2.69 | (3.20) | 59 | 1.42 | (3.98) | 22 | 3.36 | (3.29) | 21 |
| P400 Amplitude Towards-Away | -0.83 | (3.61) | 59 | 0.60 | (5.88) | 22 | -1.47 | (3.98) | 21 |

*P100* *Amplitude*: The overall model was not significant (Wald χ2 (5) = 3.23, p=.665), even after removing outlier (>3SD in model or on toward-away contrast).

*N290 Amplitude:* (Wald χ^2^ (5) = 29.86, p<.001) There was a significant effect of condition (χ2 (1) = 17.82, p<.001), but no group or interaction effect. The effect was such that the N290 amplitude was higher during the Noise versus Face condition (contrast = 4.12, SE = .80, p<.001, 95% CI [2.55, 5.69]. Removing one outlier (>3SD on face-noise contrast) did not change results.

*P400 Amplitude:* The overall model was not significant (Wald χ^2^ (5) = 6.33, p=.276) and no change when removing outliers.

# Supplement 5: Mid-Childhood WASI Scores

We repeated the analysis including the mid-childhood WASI score (not pre-registered).

The P100 latency model became marginally significant when including gender, age at infant visit, phase, non-verbal score and adding the WASI (Wald χ2 (10) = 17.96, p=.056). WASI was a significant predictor in the model (B=0.14, SE=.06, *p*=.017). There was a significant interaction effect (χ2 (2) = 6.75, p=.034). Both the No-autism and Later-autism differed from the Early-autism group (contrast Early vs No: -8.67, SE: 3.52, *p*=.014, 95% CI [-15.58, -1.76], after FDR: *p*=.028; contrast Later vs Early: 9.24, SE: 4.28, *p*=.031, 95% CI [0.85, 17.63], after FDR: *p*=.061).

For the P400 latency model, the WASI itself was a significant predictor in the model (B=0.34, SE=.17, *p*=.042), but none of the other control variables were and it did not change the overall model (Wald χ2 (10)=27.30, p=.002; group: χ2 (2)=0.266, p=.878; condition: χ2 (1)=4.45, p=.035; interaction: χ2 (2)=14.17, p=.001).

The WASI was not significant in the N290 model and did not change the results.

Adding the WASI did not affect the amplitude models or any of the associations.

# Supplement 6: Typical Likelihood Group

We re-ran the latency models including 43 typical likelihood (TL) children. Only differences between the typical likelihood group versus other groups are mentioned here.

*Latency Models:*

P100: There was a significant group by condition interaction effect (χ^2^(3) = 13.47 *p*=.004). The TL group differed from the early-autism group (contrast = 4.99, SE = 3.77p<.001).

N290: There is a significant condition (χ^2^(1) = 7.19 *p*=.007) and group by condition interaction effect (χ^2^(3) = 9.25 *p*=.026). The TL group differs from the early-autism group (contrast = -16.94, SE = 5.88, p=.004).

P400: There was a significant group by condition interaction effect (χ^2^(3) = 13.95 *p*=.003). The TL group differed from the early-autism group (contrast =14.82, SE = 9.37, *p*=.005).

*Amplitude Models:*

P400: There was a significant group by condition interaction effect (χ^2^(3) = 8.82 *p*=.032). The TL group differed from the Early-Autism group (contrast = .98, SE = 1.09, *p* =.004). The interaction effect became non-significant after removing outliers (*p*=.126).

There was no significant group by condition interaction effect for the P100 or N290 models.

| 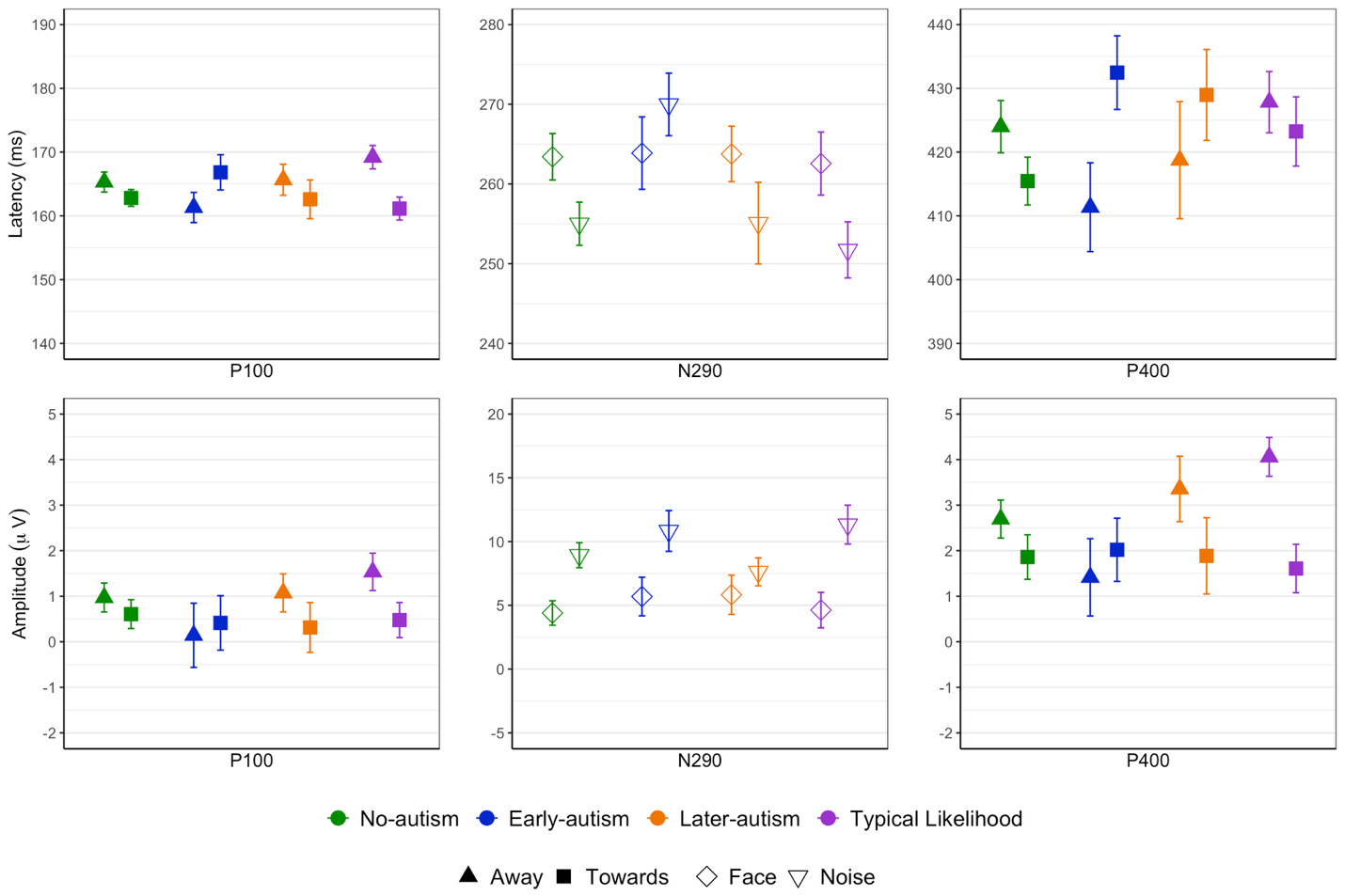 |
| --- |
| *Figure S3.* Mean and SE of latency and amplitude responses by ERP and mid-childhood outcome groups, including typical likelihood group. |

# Supplement 7: Associations with Autism traits

| Table S4: *Associations between ERP change scores and autism traits (Kendall Tau_b_ (p))* | | | | | | | | | |
| --- | --- | --- | --- | --- | --- | --- | --- | --- | --- |
|  | SRS | |  | ADOS | |  | ADI | | |
|  | SCI | RRB |  | Social Affect | RRB |  | Social | Comm. | RRB |
| P100 Latency | .097 (.180) | .016 (.831) |  | .014 (.841) | .069 (.370) |  | .101 (.153) | .045 (.528) | .041 (.587) |
| P100 Amplitude | .051 (.487) | .099 (.185) |  | .053 (.448) | .045 (.555) |  | .017 (.808) | -.044 (.532) | .069 (.353) |
| P400 Latency | **.193 (.008)** | **.214 (.004)** |  | **.151 (.032)** | **.154 (.044)** |  | .138^a^ (.050) | **.232 (.001)** | **.169 (.024)** |
| P400 Amplitude | -.002 (.983) | -.005 (.955) |  | .105 (.133) | .057 (.457) |  | -.006 (.936) | -.027 (.705) | -.037 (.622) |
| N290 Latency | -.123 (.119) | -.072 (.377) |  | -.041 (.597) | -.044 (.604) |  | **-.187 (.015)** | -.120 (.123) | **-.176 (.031)** |
| N290 Amplitude | -.035 (.657) | -.034 (.681) |  | .082 (.287) | .042 (.618) |  | .058 (.457) | .035 (.653) | .075 (.360) |
| Note. SRS = Social Responsiveness Scale; ADOS = Autism Diagnostic Observation Schedule; ADI = Autism Diagnostic Interview; SCI = Social Communication and Interaction; RRB = Restricted Interests and Repetitive Behaviour; Comm = Communication. Associations using Kendall Tau_b_  ^a^ After removing outlier: Kendall Tau_b_ = 0.151, *p* = .033 | | | | | | | | | |

# References

Elsabbagh, M., Mercure, E., Hudry, K., Chandler, S., Pasco, G., Charman, T., Pickles, A., Baron-Cohen, S., Bolton, P., & Johnson, M. H. (2012). Infant Neural Sensitivity to Dynamic Eye Gaze Is Associated with Later Emerging Autism. *Current Biology*, *22*(4), 338–342. https://doi.org/10.1016/J.CUB.2011.12.056

Haartsen, R., Jones, E. J. H., Orekhova, E. V., Charman, T., Johnson, M. H., Baron-Cohen, S., Bedford, R., Blasi, A., Bolton, P., Chandler, S., Cheung, C., Davies, K., Elsabbagh, M., Fernandes, J., Gammer, I., Garwood, H., Gliga, T., Guiraud, J., Hudry, K., … Volein, A. (2019). Functional EEG connectivity in infants associates with later restricted and repetitive behaviours in autism; a replication study. *Translational Psychiatry*, *9*(1). https://doi.org/10.1038/s41398-019-0380-2

Orekhova, E. V., Elsabbagh, M., Jones, E. J., Dawson, G., Charman, T., & Johnson, M. H. (2014). EEG hyper-connectivity in high-risk infants is associated with later autism. *Journal of Neurodevelopmental Disorders*, *6*(1), 40. https://doi.org/10.1186/1866-1955-6-40

Shephard, E., Milosavljevic, B., Mason, L., Elsabbagh, M., Tye, C., Gliga, T., Jones, E. J., Charman, T., Johnson, M. H., Baron-Cohen, S., Bedford, R., Bolton, P., Chandler, S., Fernandes, J., Garwood, H., Hudry, K., Pasco, G., Pickles, A., Tucker, L., & Volein, A. (2020). Neural and behavioural indices of face processing in siblings of children with autism spectrum disorder (ASD): A longitudinal study from infancy to mid-childhood. *Cortex*, *127*, 162–179. https://doi.org/10.1016/j.cortex.2020.02.008

Tye, C., Bussu, G., Gliga, T., Elsabbagh, M., Pasco, G., Johnsen, K., Charman, T., Jones, E. J. H., Buitelaar, J., & Johnson, M. H. (2022). Understanding the nature of face processing in early autism: A prospective study. *Journal of Psychopathology and Clinical Science*, *131*(6), 542–555. https://doi.org/10.1037/abn0000648
